# Interstitial needles versus intracavitary applicators only for locally advanced cervical cancer: results from real-life dosimetric comparisons

**DOI:** 10.3389/fonc.2024.1347727

**Published:** 2024-03-19

**Authors:** Abel Cordoba, Estelle Gesta, Alexandre Escande, Alexandra Noeuveglise, Romain Cayez, Adrien Halty, Mohamed Tahar Ladjimi, Fabrice Narducci, Delphine Hudry, Carlos Martinez Gomez, Sofia Cordoba, Marie-Cécile Le Deley, Maël Barthoulot, Eric F. Lartigau

**Affiliations:** ^1^ Department of Radiotherapy and Brachytherapy, Oscar Lambret Center, Lille, France; ^2^ Department of Radiotherapy, Centre Léonard de Vinci, Dechy, France; ^3^ Department Medical Physics, Oscar Lambret Center, Lille, France; ^4^ Department Gynecologic surgical Oncology, Oscar Lambret Center, Lille, France; ^5^ Department of Radiotherapy and Brachytherapy, Hospital Puerta de Hierro, Madrid, Spain; ^6^ Department Biostatistics and Methodology, Oscar Lambret Center, Lille, France

**Keywords:** cervical cancer, brachytherapy, radiotherapy, interstitial needles, chemotherapy

## Abstract

**Background and purpose:**

Image-guided adapted brachytherapy (IGABT) is superior to other radiotherapy techniques in the treatment of locally advanced cervical cancer (LACC). We aimed to investigate the benefit of interstitial needles (IN) for a combined intracavitary/interstitial (IC/IS) approach using IGABT over the intracavitary approach (IC) alone in patients with LACC after concomitant external beam radiotherapy (EBRT) and chemotherapy.

**Materials and methods:**

We included consecutive patients with LACC who were treated with IC/IS IGABT after radiochemotherapy (RCT) in our retrospective, observational study. Dosimetric gain and sparing of organs at risk (OAR) were investigated by comparing the IC/IS IGABT plan with a simulated plan without needle use (IC IGABT plan) and the impact of other clinical factors on the benefit of IC/IS IGABT.

**Results:**

Ninety-nine patients were analyzed, with a mean EBRT dose of 45.5 ± 1.7 Gy; 97 patients received concurrent chemotherapy. A significant increase in median D90% High Risk Clinical target volume (HR-CTV) was found for IC/IS (82.8 Gy) vs IC (76.2 Gy) (p < 10^-4^). A significant decrease of the delivered dose for all OAR was found for IC/IS vs IC for median D2_cc_ to the bladder (77.2 Gy), rectum (68 Gy), sigmoid (53.2 Gy), and small bowel (47 Gy) (all p < 10^-4^).

**Conclusion:**

HR-CTV coverage was higher with IC/IS IGABT than with IC IGABT, with lower doses to the OAR in patients managed for LACC after RCT. Interstitial brachytherapy in the management of LACC after radiotherapy provides better coverage of the target volumes, this could contribute to better local control and improved survival of patients.

## 1 Introduction

Cervical cancer accounted for more than 500,000 new cancer cases worldwide in 2018, and remains the third leading cause of cancer-related deaths among women in developing countries (1). Over 66,000 women are diagnosed with cervical cancer every year within the World Health Organization European region, and over 30,000 die from this preventable disease (2). Over the last 30 years, a decrease in the incidence and mortality of cervical cancer has been observed in developed countries (North America and Western Europe) owing to the democratization of screening and the development of vaccination of young women between the ages of 12 and 26 years against Human Papilloma Virus, particularly against HPV-16 and HPV-18 (3).

Concomitant radiochemotherapy (RCT) and image-guided adapted brachytherapy (IGABT) are the main treatment for locally advanced cervical cancer (LACC) (4). IGABT is superior to other radiotherapy techniques for the treatment of LACC (5). IGABT comprises both the implantation of MRI/CT in contouring tools to delineate residual tumor accuracy (6) and the use of intracavitary interstitial applicators and interstitial needles (IC/IS) to adapt brachytherapy material to each patient (7), which has allowed the delivery of higher doses to residual tumors while sparing the organs at risk (OAR).

The clinical benefits of IGABT, mostly in terms of local control, have been published in large retrospective series (8–12) and recently in the EMBRACE prospective protocol (13). The use of interstitial needles has been democratized and is now established as a quality indicator according to the GEC-ESTRO group in teams performing brachytherapy in LACC (14). The main dosimetric prognostic factor for local control is the dose delivered to the residual tumor and the whole cervical tissue, called the high-risk clinical target volume (HR-CTV) (8, 10, 12, 13). We aimed to investigate the benefit of interstitial needles for combined IC/IS IGABT versus IC IGABT alone for patients with LACC.

## 2 Materials and methods

### 2.1 Study design

We conducted a single-center, retrospective, observational study in which all patients were consecutively included as follows: LACC patients, > 18 years of age, treated with intracavitary/interstitial (IC/IS) brachytherapy after external beam radiotherapy treatment (EBRT); between January 2017 and December 2020; with a diagnostic pelvic MRI and an MRI at first IGABT application. The exclusion criteria were incomplete RCT and IGABT treatment, noncervical primitive tumors, absence of MRI during IGABT, and refusal of consent. Patient, tumor, treatment, dosimetric, and outcome characteristics were collected from patient medical records. All patients were clinically staged according to International of FIGO criteria (15). Clinical tumor size was defined as the maximum size on clinical examination or MRI in centimeters.

The included patients did not object to the use of their clinical data for research purposes, and the study complied with the Reference Methodology MR004 adopted by the Commission Nationale de l’Informatique et des Libertés (CNIL).

### 2.2 Treatment

All patients were treated for LACC using radiotherapy (RT) or RCT and IC/IS IGABT. EBRT consisted of 25 or 28 fractions (1.8 Gy to deliver 45 or 50.4 Gy to the pelvis and/or para-aortic lymph nodes, if indicated and 2.2 or 2.4 Gy to deliver 55 or 60 Gy positive lymph node involvement suspected either from positron emission tomography or MRI or after lymph node staging). Concomitant platinum chemotherapy was prescribed to all patients either by weekly cisplatin 40 mg/m^2^ or carboplatin AUC 2. IGABT was performed immediately after the completion of RT or RCT.

Before brachytherapy, an MRI with contrast was performed at the end of RCT to determine early tumor response to primary treatment. IGABT (four high dose rate fractions of 6.5-7Gy) consisted of one or two applications under general anesthesia; a Utrecht applicator (Nucletron®, Veenendaal, The Netherlands) with vaginal ovoids was used. After visualization of MRI at the end of RT/RCT and clinical examination, we proceeded to placement of the adapted applicators in the surgical room (14). After cervix dilatation, the intracavitary and ovoid applicators were adapted to the patient and tumor, and ultrasound control-guided interstitial implants were placed for each ovoid (3 laterals, 1 anterior, 1 posterior) depending on the residual tumor seen by MRI and clinical examination. Only needles parallel to the endo-uterine catheter were implanted, and there were no possibility to implant obliques needles due to the use of the Utrecht applicator. Finally, the vagina and packed to fix the applicator.

All patients underwent an MRI with the applicator in place to delineate the gross tumor volume (GTV), HR-CTV, IR-CTV, rectum, sigmoid, bladder, and small bowel. The prescription dose for high resolution CT (HRCT) was 6.5–7 Gy per fraction, for a total of four fractions. We aimed to deliver at least an equivalent dose of 2 Gy per fraction (EQD2) of 85 Gy to the D90 HR-CTV. Using the Raystation treatment planning system, HR-CTV and OAR were contoured from MRI brachytherapy images on the day of the first implantation using information from both the clinical examination and MRI-image, according to the GYN-GEC-ESTRO recommendations (6).

### 2.3 Simulated treatment and treatment plan

We used the EMBRACE protocol dosimetric requirements (16) based on GEC-ESTRO-ABS recommendations for CTVs and OARs in EQD2: D_90 HR-CTV_ had to be more than 85 Gy, without compromising OAR (D_2cc_ of bladder, rectum, and sigmoid less than 90 Gy, 75 Gy, and 75 Gy, respectively). The linear-quadratic model with α/β = 10 Gy for tumor and α/β = 3 Gy for OAR was used to evaluate total dose reported during EBRT and IGABT. We considered that the OAR received all prescribed doses during EBRT. Intermediate-risk CTV (IR_CTV), GTV, and ICRU reference points (rectovaginal point, bladder point, vaginal point, PIBS, PIBS-2, and Point A) were reported.

According to the initial plan, we created a simulated treatment, deactivated interstitial positions, and re-optimized with IC only to deliver the highest possible dose to the HR-CTV according to the OAR dose limitation.

### 2.4 Statistical analysis

The characteristics of the population are described in terms of median and range for quantitative data, and in terms of frequency and percentage for qualitative data. Different dosimetric parameters were estimated for each approach (median, range) and compared between the two treatment plans using Student’s t-test for paired samples or Wilcoxon’s signed rank test for paired samples when the hypotheses of Student’s t-test were not valid (normal distribution of the quantitative variable, equality of the variances in the two groups). The dosimetric data have also been treated as qualitative variables and compared using the McNemar’s test for paired sample. The relationship between the benefit of IN and FIGO 2018 stage, initial tumor size, laterality of affected parameters, number of IN placements, and period of IN placement were explored using univariate logistic regression models.

## 3 Results

**Figure 1:**
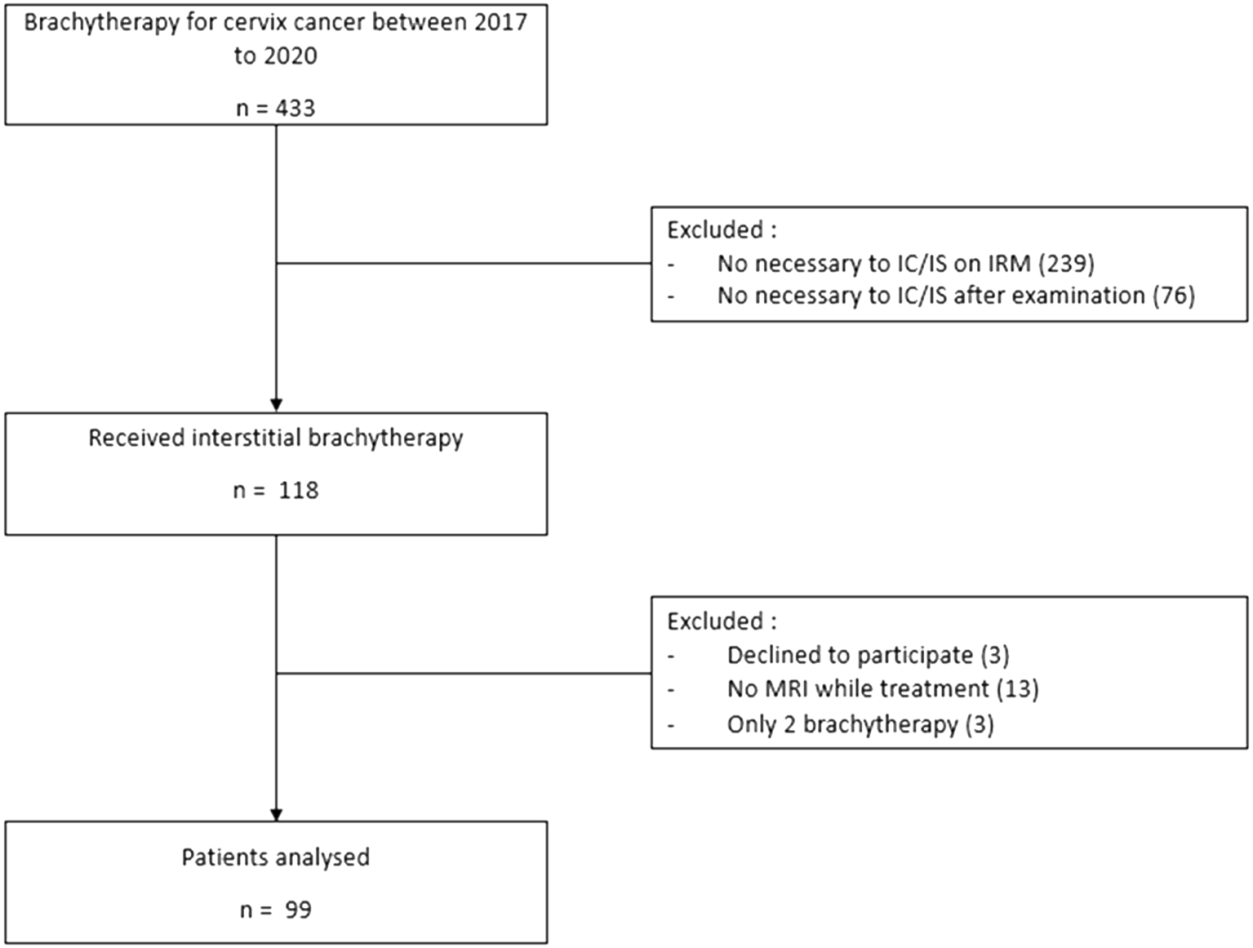
Flow chart of the study.

A total of 194 patients were treated for LACC with IGABT after RCT between 2017 and 2020. A total of 76 patients were excluded because they were treated with IC brachytherapy alone. Thirteen patients were excluded because brachytherapy MRI was not performed due to contraindications to MRI (seven patients) or unavailability (six patients); three patients received incomplete brachytherapy treatment; and three patients opposed the re-use of their data, leaving 99 patients for analysis (**Figure 1**).

The median age at diagnosis was 51 years (range, 23–86). Five patients had been treated for cervical cancer relapse, with three patients treated after total hysterectomy. Eighty-two (82.8%) patients had squamous cell carcinoma. Mean EBRT dose was 45.5 ± 1.7 Gy; 97% received concurrent chemotherapy. Mean HR-CTV was 40 ± 19.1 cm^3^; 63 (68.5%) patients had an HR-CTV ≥30 cm^3^. At diagnosis, parametrial invasion was observed in 92 (92.9%) patients, bilateral parametrial invasion in 66 (66.7%), and distal parametrial invasion in 41 (41.4%). Seven patients with FIGO stage IVB tumors were treated with RCT and IGABT after first-line chemotherapy. Seventy-seven (77.7%) patients underwent MRI at the end of the RCT before brachytherapy. The clinical characteristics of the patients are shown in **Table 1**.

**Table 1:** Quantitative variables are described using median (range). Qualitative variables are described using number (percentage).

	TOTAL
	N = 99
Patients	
Age (years)	51 (23–86)
OMS	
0–1	96 (96.9%)
2–3	3 (3.1%)
Histology	
SCC	82 (82.8%)
Adenocarcinoma	17 (17.2%)
MRI initial (MD=1)	
Volume (cm^3^)	83.6 (53–76)
Parametrial invasion (PI)	92 (93.9)
Bilateral PI	66 (71.7)
Distal PI	41 (44.6)
Stage FIGO 2018	
IIA1	1 (1%)
IIA2	2 (2%)
IIB	11 (11.1%)
IIIB	6 (6.1%)
IIIC1	24 (24.2%)
IIIC2	28 (28.3%)
IVA	20 (20.2%)
IVB	7 (7.1%)
EBRT and CT	
EBRT dose	
Volume EBRT	
Pelvic	53 (53.5%)
Pelvic + PAN	46 (46.5%)
Concurrent chemotherapy	96 (97%)
MRI 45 Gy (MD=12)	
Volume	24.2 (0–111)
*Brachytherapy*	
Number of IS/IC	
<4	57 (57.6%)
>=4	42 (42.4%)
2017–2018	19 (19.2%)
2019–2020	80 (80.8%)
Volume HR-CTV (cm^3^)	35 (9.7–103)
Volume HR-CTV ≥ 30 cm^3^	63 (68.5)
Response to EBRT	
> 1.1	21 (21.2)
0.9–1.1	2 (0.1)
< 0.9	66 (66.7)
Treatment time (days)	55 (50–62)

Quantitative variables are described using mean (range). Qualitative variables are described using number (percentage).

SCC, Squamous Cell Carcinoma; PAN, ParaAortic Node; IN, Interstitial needle, MRI, magnetic resonance imaging, EBRT, external beam radiotherapy, CT, Chemotherapy, IS/IC interstitial and intracavitary brachytherapy

Different dosimetric parameters were compared between the IC/IS IGABT plan and the simulated plan without needle use (IC IGABT plan) (**Table 2**). A significant increase in median D90% HR-CTV was found (IC/IS vs IC: 82.8 Gy [56.4–92.3 Gy] vs 76.2 Gy [54.4–97.8 Gy], p<10^-4^). A significant decrease of the delivered dose for all OAR was found (IC/IS vs IC): median D2cc for the bladder was 77.2 (53.2–90.4 Gy) vs 85.2 Gy (60.2–91.7 Gy), p<10^-4^; median D2cc for the rectum was 68 Gy (48.1–96.2 Gy) vs 72.7 Gy (49.5–80.4 Gy), p<10^-4^; median D2cc for the sigmoid was 53.2 Gy (44.9–70.5) vs 53.5 Gy (45.1–80.3 Gy), p<10^-4^; and median D2cc for the small bowel was 47 Gy (43.9–68.2 Gy) vs 47.5 Gy (44–74.6 Gy), p<10^-4^.

**Table 2:** Dosimetric analysis.

	IC/IS	IC	p-value
Target volume (Gy)			
D_98%_ GTVres	78.7 (53.7–99.0)	71.0 (53.1–109.5)	< 10^-4^
D_98%_ HR-CTV	72.1 (52.4–83.7)	67.1 (51.9–83.5)	< 10^-4^
D_90%_ HR-CTV	82.8 (56.4–92.3)	76.2 (54.4–97.8)	< 10^-4^
D_90%_ HR-CTV ≥85 Gy (%)	38 (38.4)	19 (19.2)	< 10^-4^
D_98%_ CTV-RI	56.1 (48.9–62.3)	56.2 (48.5–65.0)	0.746
D_90%_ CTV-RI	59.6 (50.7–65.1)	59.8 (50.2–68.7)	0.812
OAR (Gy)			
D2_cc_ Bladder	77.2 (53.2–90.4)	85.2 (60.2–91.7)	< 10^-4^
D2_cc_ Bladder < 80 Gy (%)	66 (66.7)	27 (27.3)	< 10^-4^
D2_cc_ Rectum	68.0 (48.1–96.2)	72.7 (49.5–80.4)	< 10^-4^
D2_cc_ Rectum < 65 Gy (%)	32 (32.3)	17 (17.2)	< 10^-3^
D2_cc_ Sigmoid	53.2 (44.9–70.5)	53.5 (45.1–80.3)	< 10^-4^
D2_cc_ Sigmoid < 70 Gy (%)	98 (99.0)	94 (94.9)	0.046
D2_cc_ Bowel	47.0 (43.9–68.2)	47.5 (44.0–74.6)	< 10^-4^

Quantitative variables are described using median (range) and qualitative variables are described using number (percentage). Comparison between groups was performed using Wilcoxon signed rank test for paired samples (quantitative variables) or McNemar test for paired sample (qualitative variables). GTV res, residual (res) volume of gross tumor volume at the time of brachytherapy

**Figure 2:**
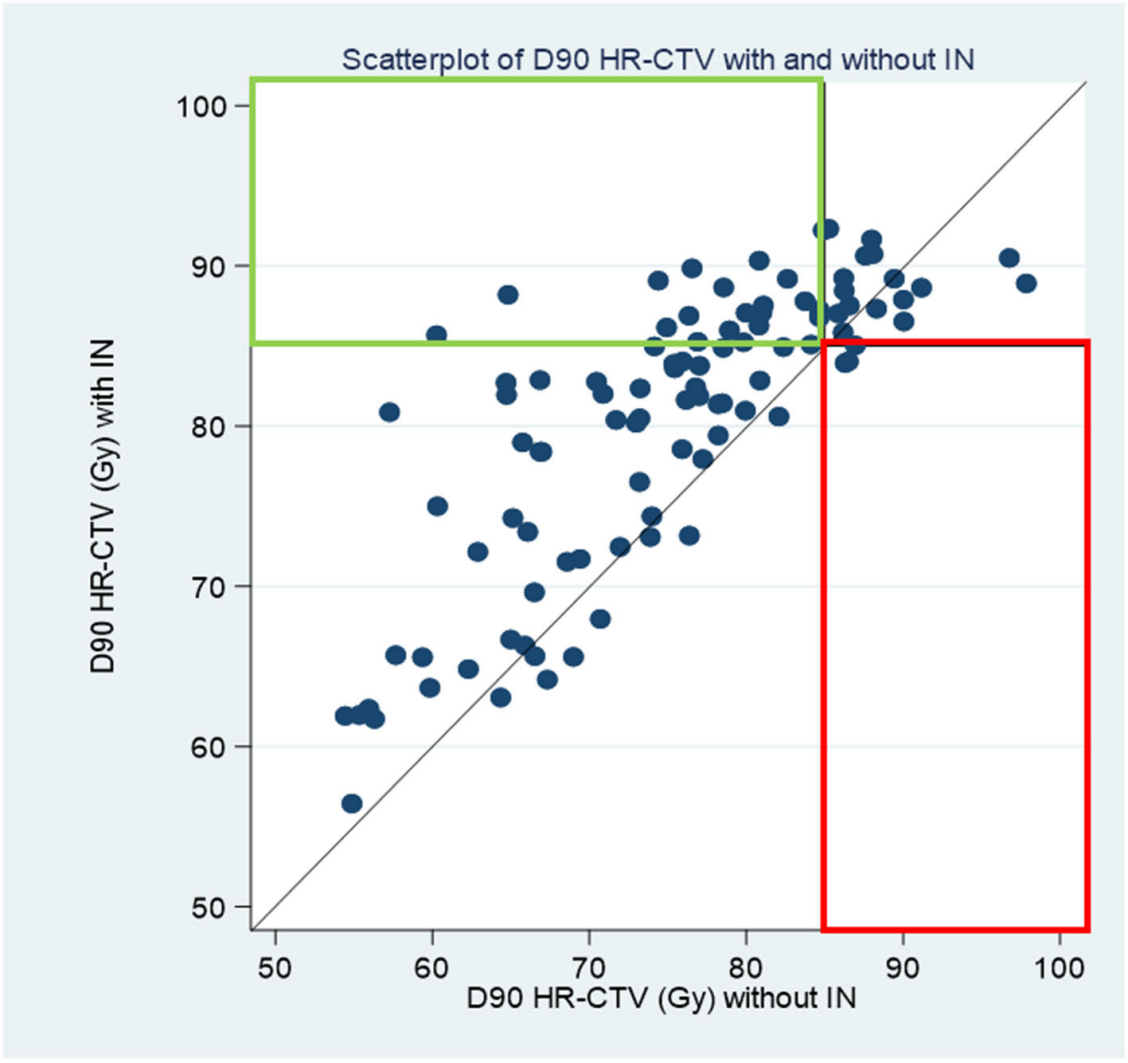
scatterplot of D90 HRCTV with IC IS versus D90 HRCTV with IC.

**Figure 3:**
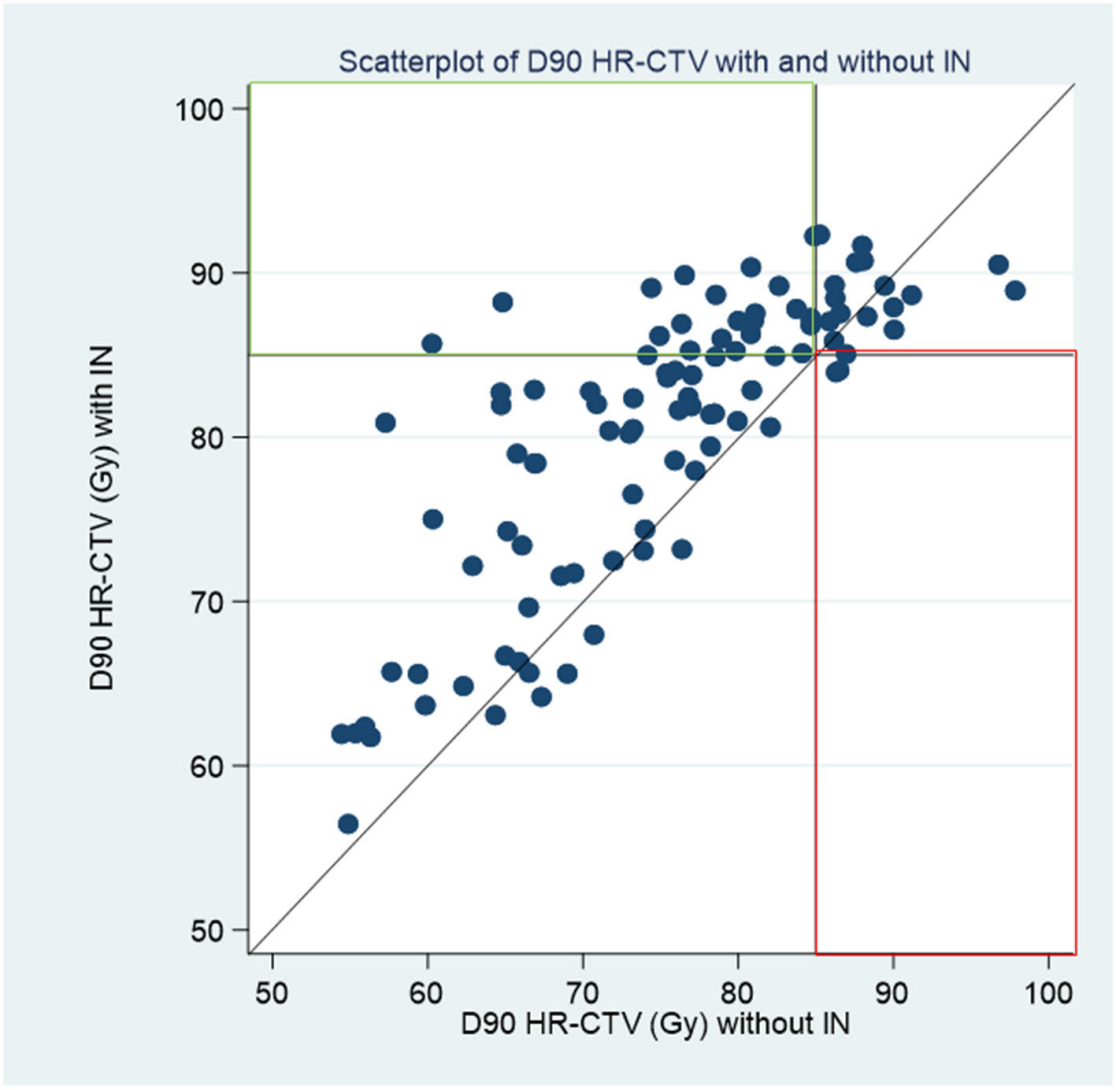
IC/IS HR-CTV D90 versus IC HR-CTV D90.

**Figure 2** shows a scatterplot of D90% HR-CTV with IC/IS (ordinate) versus D90% HR-CTV with IC (abscissa). This graph shows the patients whose D90% HR-CTV with IC/IS was better than that with IC; these patients were above the bisector. Moreover, if we define good coverage as a D90% HR-CTV > 85 Gy, this graph shows that 21 (21.2%) patients benefited from needle insertion (green rectangle), while two (2.0%) patients were penalized (red rectangle). For the remaining 66 (66.7%) patients, there was no impact and coverage was good with or without the IC plan using a needle. **Table 3** describes the different situations observed from **Figure 3**: the percentage of HR-CTV couverture depending on IC or IC/IS implantation.

**Table 3:** Percentage of couverture depending on IC or IC/IS implantation.

With IC plan With IS/IC plan	Bad coverage n	Good coverage n	Total n
**Bad coverage**	**59**	**2 (2%)**	**61**
**Good coverage**	**21 (21%)**	**17**	**38**
**Total**	**80**	**19**	**99**

Using simple logistic regression models, we investigated whether there was a relationship between the benefit of IC/IS and FIGO 2018 stage, initial tumor size, laterality of affected parameters, number of needles placed, and period of needle placement, respectively (**Table 4**). There was a statistically significant relationship between the benefit of IC/IS versus IC alone and the laterality of the affected parameters (p = 0.05); however, this result should be interpreted with caution as the numbers are very small.

**Table 4:** Factors explaining the difference in HR-CTV D90 (IC/IS vs IC).

Variables	Coefficient	IC 95%	p
Stade FIGO	0.55	-0.93-2.04	0.461
Tumor volume	-0.01	-0.07-0.06	0.825
Laterality	-1.27	-4.09-1.56	0.376
Number of needles	**1.21**	**0.25-2.18**	**0.014**
**Period (2017-2018 vs 2019-2020)**	**6.16**	**3.21-9.11**	**<0.001**

## 4 Discussion

We performed this retrospective analysis of the first 99 patients treated at our institution using IGABT with IC/IS after RT or RCT for LACC. Our results showed a benefit of using interstitial needles during uterovaginal brachytherapy on target volume coverage, particularly in high-risk CTV (HR-CTV), in the management of LACC after RT or RCT. Indeed, the application of interstitial needles during uterine brachytherapy delivered 85 Gy on the D90 HR-CTV in 38.4% of patients, compared to only 19.2% if we had performed endo-uterine brachytherapy with IC plan interstitial needles. To interpret these results from the point of view of a new technique developed in our Brachytherapy Unit, we started performing interstitial implants in January 2017 and, at that time, our only applicator was the Utrecht. Therefore, we only had the possibility of implanting interstitial needles parallel to the endo-uterine probe, with no possibility of performing implants with oblique needles.

Mazeron et al. (10) showed in their series of 225 consecutively treated patients that the goal of administering > 85 Gy to the HR-CTV was only possible in 30.7% of the cases; in their series, there were no data on the percentage of patients treated with IGABT IC/IS. In our series, we identified only 21% of patients who benefited from IC/IS IGABT and, for 77% of patients, there was no benefit of adding IC/IS to IC alone, which can be explained by the learning process of the medical team, poor patient selection, and the inability to perform only IGABT implants with oblique needles to cover the disease in the distal parametria. Indeed, the interest in performing simulated treatment with IC/IS before starting brachytherapy was studied in 58 patients (17). The application of IC plan interstitial needles followed by dosimetric MRI was performed before brachytherapy. Finally, only 41% of patients received treatment with interstitial needles. Tumors eccentric to the endo-uterine probe and OAR close to the target volumes were good indications for brachytherapy with IC/IS. Furthermore, the D90 for HR-CTV was higher than 85 Gy EQD2 in all patients with the IC/IS preplan and only in 50% of the patients using the optimized IC preplan. The dose volume histogram constraints for OAR were respected in 79% of the IC/IS plans compared with 46% for the optimized IC preplan.

The first series of interstitial needles improved the dose in the HR-CTV. The RETRO EMBRACE series (18) retrospectively analyzed 610 patients treated by RCT and IGABT for LACC; the team investigated the evolution of IC brachytherapy towards IC/IS over time and proved that D90 HR-CTV EQD2 is superior when using IGABT with IC/IS (92 Gy ± 13 Gy) compared to IC alone. Similarly, the role of interstitial brachytherapy and the use of interstitial oblique and parallel needles were reported in 2019 (19). In 69 patients with longer residual tumor after RCT (HR-CTV volume: 69 ± 32 cm^3^), they showed that the D90 HR-CTV of 86 ± 7 Gy with cumulative mean EQD2 for the bladder, rectum, and sigmoid D2 cm^3^ was 86 ± 12 Gy, 68 ± 7 Gy, and 68 ± 9 Gy, respectively. In their series, they implanted a median of seven [3–15] needles with four oblique needles [1–7].

Rogowski et al. (20) published their series of 44 patients treated consecutively for LACC by RCT and IC/IS IGABT with the Venezia applicator. They showed that the D90 HR-CTV of 92.3 Gy EQD2 (72.2–100.8) with D_2cc_ EQD2 for the bladder, rectum, sigmoid, and bowel were 74.8 Gy (58.6–89.7), 57.9 Gy (49.6–72.4), 60.0 Gy (47.2–75.0), and 52.4 Gy (44.1–72.1), respectively. This series illustrates how the use of oblique interstitial needles allows coverage of the target volume by decreasing the dose to organs such as the bladder, rectum, and sigmoid colon.

Our study proposes a design with a direct comparison of two treatment plans with and without IC interstitial needles during uterovaginal brachytherapy in a large population, which allows an adapted evaluation for each patient. We showed a correlation between the HR-CTV volume and the probability of needle implantation. It is important to note that, during the study period, we used an Utrecht applicator with the possibility of inserting only interstitial needles parallel to the endo-uterine probe, without the possibility of inserting oblique needles for more distal parametrial infiltrations.

There is also a factor linked to the period in which the implants were placed and HR-CTV coverage. Our results in terms of HR-CTV coverage were better in the period of 2019–2020 than in 2017–2018. This effect can be explained by several factors, but is probably due to a better selection of patients and better acknowledgement of the IC/IS technique.

We chose to conduct the analyses on the target volumes, with the only limit being the maximum acceptable dose received in nearby OAR. The D_2cc_ EQD2 delivered to the bladder (75.5 vs 81.1 Gy), rectum (67.2 vs 70.2 Gy), and sigmoid (53.8 Gy vs 55 Gy) are, therefore, higher than for brachytherapy with interstitial needles, and the analyses performed are, therefore, not interpretable in view of the study design.

Our study had some limitations. We observed poorer coverage of HR-CTV, explained by several reasons. It is our experience that, in the treatment of patients with larger tumors using the IC/IS IGABT technique and learning time from initiation of a new technique, obtaining expected results is important, and eligibility of patients to IC/IS may be difficult. We also initiated this technique with an Utrecht applicator that gives you the opportunity to place interstitial needles only in the same axis of the intrauterine tandem; we did not place oblique needles for patients who presented with more distal parametrial infiltration.

## 5 Conclusion

The use of interstitial brachytherapy in the management of LACC after radiotherapy provides better coverage of the target volumes, this could contribute to better local control and improved survival of treated patients. This study reflects the real-life treatment of patients with initial tumors and a poor prognosis. The recent use of brachytherapy with interstitial needles in these patients has resulted in better tumor coverage, and the experience developed by the interventional team could also be a factor in better local tumor control.

## Data availability statement

The raw data supporting the conclusions of this article will be made available by the authors, without undue reservation.

## Ethics statement

The studies involving humans were approved by Centre Oscar Lambret CEC, Lille, France. The studies were conducted in accordance with the local legislation and institutional requirements. Written informed consent for participation was not required from the participants or the participants’ legal guardians/next of kin because The included patients did not object to the use of their clinical data for research purposes, and the study complied with the Reference Methodology MR004 adopted by the Commission Nationale de l’Informatique et des Libertés (CNIL).

## Author contributions

AC: Conceptualization, Methodology, Project administration, Resources, Validation, Writing – original draft, Writing – review & editing. EG: Methodology, Writing – original draft, Writing – review & editing. AE: Conceptualization, Methodology, Validation, Writing – review & editing. AN: Validation, Writing – review & editing. RC: Formal analysis, Writing – review & editing. AH: Formal analysis, Writing – review & editing. ML: Formal analysis, Writing – review & editing. FN: Writing – review & editing. DH: Writing – review & editing. CMG: Writing – review & editing. SC: Validation, Writing – original draft, Writing – review & editing. M-CLD: Data curation, Formal analysis, Methodology, Software, Writing – review & editing. MB: Data curation, Formal analysis, Methodology, Software, Writing – review & editing. EL: Conceptualization, Writing – review & editing.
